# Comparison of Surgical Treatment Results of Large Incisional Hernias

**DOI:** 10.7759/cureus.32020

**Published:** 2022-11-29

**Authors:** Ercan Korkut, Nurhak Aksungur, Necip Altundaş, Salih Kara, Ferdi Cambaztepe, Rıfat Peksöz, Kamber Kaşali

**Affiliations:** 1 General Surgery, Atatürk University Faculty of Medicine, Erzurum, TUR; 2 Biostatistics, Atatürk University Faculty of Medicine, Erzurum, TUR

**Keywords:** incisional hernia, incisional hernia repair, complications, open intraperitoneal sublay dual mesh method, laparoscopic intraperitoneal sublay mesh method, open onlay prolene mesh method, large incisional hernia repair

## Abstract

Introduction

Incisional hernias are one of the most common complications after abdominal surgery. Surgical repairs of large incisional hernias have higher complications and recurrence rates compared to smaller incisional hernia repairs. For this reason, it is a more difficult and experience-requiring application for surgeons. In addition, there is no evidence-based consensus in the literature regarding the optimal surgical treatment of large incisional hernias. The aim of this study is to compare the results of the three most common surgical treatment methods used in a tertiary university hospital for the repair of large incisional hernias in terms of patients' characteristics, recurrence, and complication rates of the treatment methods.

Methods

Between 2014 and 2020, 366 patients with incisional hernias with facial defects larger than 10 cm in a tertiary medical faculty hospital located in eastern Turkey were analyzed. Patients were divided into three groups according to the surgical method used: open onlay prolene mesh (OPM) method, laparoscopic intraperitoneal sublay dual mesh (IPSDM) method, and open IPSDM method. Postoperative complications were divided into five groups as follows: wound complications, complications due to surgical procedures, medical complications, recurrences, and mortality. Treatment methods were compared according to the demographic characteristics of the patients and the postoperative complication rates.

Results

Of the patients, 141 were male and 225 were female, and the mean age was 58.0 ± 28 years. Of the patients, 81.9% were operated on with the open OPM, 10.9% with the laparoscopic IPSDM, and 7.1% with the open IPSDM. Wound complications occurred in 26.7% of patients, surgical complications in 3.2%, medical complications in 6.5%, recurrence in 9.2%, and mortality in 0.8% of patients. Total wound complications were significantly higher in the open OPM group (30%) (p = 0.009). Total surgery complications were significantly higher in the laparoscopic IPSDM group (15%) (p = 0.002). There was no significant difference between groups for medical complications (p = 0.540). Although no recurrence was observed in the open IPSDM group, no significant difference was observed between the groups (p = 0.099). There was no difference in mortality rates between the groups (p = 0.450). The overall complication rate was highest in the open OPM group (48.3%) and lowest in the open IPSDM group (27%) (p = 0.092). The operative time was found to be significantly shorter in open IPSDM (p < 0.001). The length of hospital stay was highest in the open OPM group and lowest in the open IPSDM group (p = 0.450).

Conclusions

Although hernia defect is greater in the open IPSDM compared to other methods, this method is more advantageous in terms of the complication rate associated with the surgical procedure, the overall complication rate, the duration of surgery, and the recurrence rate. Laparoscopic IPSDM is a more advantageous method in terms of the overall wound and medical complications.

## Introduction

Incisional hernias occur in approximately 11-23% of patients undergoing abdominal surgery. However, this rate increases to 35% in high-risk patients [1-3]. Post-laparotomy incisional hernias are associated with inadequate healing of the abdominal fascia. Inadequate fascia healing can be due to many factors, such as the surgical technique and the patient's biological factors. In the studies of literature, the risk factors that are effective in the development of incisional hernia are stated as advanced age, obesity, BMI ≥ 35 kg/m2, diabetes, hernia size > 15 cm, American Society of Anesthesiologists (ASA) ≥ 3, chronic lung disease, respiratory failure, respiratory complications, active smokers, need for ICU cares, wound infection, patients who underwent emergency surgery for intestinal strangulation, concomitant bowel surgery, patients who have received chemotherapy, the use of glucocorticosteroids, anemia, and hypoalbuminemia. However, it has been stated that the most important risk factor is wound infection [4,5].

According to the European Hernia Society (EHS) classification, incisional hernias are divided into the following groups: (a) small (<5 cm wide or long), (b) medium (5-10 cm wide or long), and (c) large (>10 cm wide or long) [6]. However, researchers have referred to hernias with a transverse diameter of ≥15 cm as giant hernias [2,7,8]. Of all patients with an incisional hernia, 15-47% have a large incisional hernia (LIH), of which approximately 11% are giant incisional hernias [1,2,9].

Surgical management of incisional hernias is a major challenge for surgeons due to the high complication rate. In studies of LIH, the morbidity rate ranges from 4% to 100% (median: 32%), the recurrence rate ranges from 0% to 35% (median: 5-8.3%) after a follow-up period of one to five years, and the mortality rate ranges from 0% to 5% (median: 0-2%) [1,2,10]. Surgical treatment of LIH is more difficult than for small and medium-sized hernias and requires experience. It is recommended that experienced surgeons perform the treatment of LIH [1,11]. The choice of optimal surgical technique in treating LIH is controversial, and the number of high-level evidence-based papers is limited [2,12].

Surgical site infection, recurrence, mesh infection, wound dehiscence, seroma, and bowel injury are complications reported in the literature following incisional hernia repair. Among them, wound infection is the most common. The fact that the patient has comorbid factors and a large ventral hernia complicates the management of LIH [10-13].

It is recommended that mesh be used in the surgical treatment of incisional hernias larger than 2 cm because of the lower recurrence rate [8,14,15]. In the primary closure of ventral hernias, the recurrence rate is up to 60%, while this rate is 10-30% for repairs with mesh [1,3,16].

Surgical methods are referred to as onlay, inlay, or sublay, depending on the anatomic position in which the mesh is placed. In the onlay position, the mesh is fixed by placing it on the anterior surface of the rectus muscle and fascia. In the inlay position, also known as interposition bridging mesh, the mesh is fixed to the edges of the fascial defect. In the sublay position, the mesh is fixed behind the rectus muscle and abdominal fascia. In the sublay position, the mesh is attached either supraperitoneally or retroperitoneally (within the abdominal cavity) to the anterior surface of the peritoneum [1].

This study aims to compare the results of the three most commonly used surgical techniques for LIHs and to discuss them in light of the current literature.

## Materials and methods

The approval for the study was obtained from the Ethics Committee of Erzurum Atatürk University Faculty of Medicine (No.: 02.06.2022/B.30.2.ATA.0.01.00/464).

A total of 601 adult patients who underwent surgery for incisional hernia between January 2014 and December 2020 were retrospectively analyzed in a tertiary medical faculty hospital located in eastern Turkey. According to the EHS definition, hernias with a facial defect of 10 cm or more in any direction were defined as LIH. Patients over 18 years of age with a fascial defect greater than 10 cm in any direction were included in the study. Patients undergoing emergency surgery, patients with bowel perforation, intra-abdominal infection, an ASA score greater than 3, and patients who underwent rare hernia repair methods were not included in the analysis. A total of 366 patients were included in the study. The surgeries were performed by general surgeons experienced in LIH.

Depending on the surgical method used, patients were divided into three groups: the open onlay prolene method (OPM; n = 300), the laparoscopic intraperitoneal sublay dual mesh (IPSDM) method (n = 40), and the open IPSDM method (n = 26).

Patients with postoperative complications were divided into five groups. Group 1 was defined as wound complications, group 2 as surgical-related complications, group 3 as medical complications, group 4 as patients with recurrence, and group 5 as patients with mortality.

Propylene mesh was used in the open OPM technique and dual-composite mesh was used in the laparoscopic IPSDM and open IPSDM techniques. Preoperative antibiotic prophylaxis (cefazolin sodium) was administered to all patients. Deep vein thrombosis prophylaxis and antibiotic prophylaxis were given to all patients. The bladder and stomach were catheterized. Discharged patients were monitored on day 10 and day 30. Patients without symptoms were called for follow-up after one year.

Data on age, sex, BMI, duration of operation, length of hospital stay, postoperative complications, recurrence, and mortality were statistically compared.

Statistical analysis

SPSS version 26 software (IBM Corp., Armonk, NY) was used for statistical analysis. Quantitative parameters were expressed as arithmetic mean ± standard deviation, and categorical variables were expressed as numbers and percentages. The distribution of numerical data was assessed with the Shapiro-Wilk test, the Kolmogorov-Smirnov test, and histogram graphs. Nonparametric tests were performed for data that did not show normal distribution. The chi-square test was used to compare categorical data. Data were analyzed with a 95% confidence interval, and p < 0.05 was accepted as statistically significant.

## Results

Of the patients, 141 were male and 225 were female, and the mean age was 58.0 ± 28 (range: 24-82) years. The mean BMI was 31.6 ± 15 (range: 20-52). The mean diameter of the fascial defect was 13.6 ± 9.8 (range: 10.5-30) cm. The mean operative time of the patients was 115 + 52 minutes, and the hospital stay was 5.8 ± 5 days. The mean follow-up time of patients was 36 ± 25 months (20-70) (Table 1).

**Table 1:** Patients' demographic features and clinical characteristics OPM, onlay prolene method; IPSDM, intraperitoneal sublay dual mesh.

Variables	Mean ± SD	Open OPM (n = 300)	Laparoscopic IPSDM (n = 40)	Open IPSDM (n = 26)	P-value
Age (years)	58.0 ± 28	58 ± 25	56 ± 19	62 ± 25	0.618
Sex					
Male	141	115	15	11	0.914
Female	225	185	25	15	
BMI (kg/m^2^)	31.6 ± 15	31.8 ± 10	30.1 ± 7.5	32 ± 15	0.603
Defect diameter (cm)	13.6 ± 9.8	13.3 ± 5.5	11.3 ± 2.5	22 ± 7.5	<0.001 (1-3, 2-3)
Operation time (min)	115 ± 52	116 ± 25	125 ± 45	90 ± 30	<0.001 (1-3, 2-3)
Length of stay/day	5.8 ± 5	6.1 ± 7	5.3 ± 7	4.5 ± 1.5	0.430
Average follow-up time (months)	36 ± 25	38 ± 26	33 ± 23.5	33 ± 24.5	0.358

A total of 300 (81.9%) patients were operated on with the open OPM method, 40 (10.9%) patients with the laparoscopic IPSDM method, and 26 (7.1%) patients with the open IPSDM method.

In terms of hernia diameter by groups, the hernia defect diameter was 13.3 ± 5.5 cm for the open OPM method, 11.3 ± 2.5 cm for the laparoscopic IPSDM method, and 22 ± 7.5 cm for the open IPSDM method. The larger defect diameter with the open IPSDM method was statistically significant compared to both treatment methods (p < 0.001). There was no statistically significant difference between treatment groups in age, sex, and BMI (kg/m2) (p > 0.005).

Wound complications occurred in 98 (26.7%) patients in group 1. The distribution by the group was 90 patients (30%) in open OPM, four patients (10%) in the laparoscopic IPSDM method, and four patients in the open IPSDM (15.3%). Higher wound complications in the open OPM method compared to the other two groups were statistically significant (p = 0.009).

Complications related to the surgical procedure occurred in 12 (3.2%) patients. Surgical complications occurred in six patients (2%) in the open OPM method and six patients (15%) in the laparoscopic IPSDM, and no complications related to the surgical procedure were observed in the open IPSDM. The rate of surgical complications in the laparoscopic IPSDM method was statistically significant compared to the other two groups (p = 0.002). In addition, early detachment of the mesh after surgery was statistically significant in two patients in the laparoscopic IPSDM method compared with the other methods (p = 0.017).

Medical complications occurred in a total of 24 (6.5%) patients. Complications occurred in 19 (6.3%) patients with open OPM, in two (5%) patients with laparoscopic IPSDM, and in three patients (11.5%) with open IPSDM. There was no statistically significant difference between the groups in medical complications (p = 0.540).

Recurrence was observed in 34 patients (9.2%). Recurrence was observed in 28 patients (9.3%) in the open OPM method, and six patients (15%) in the laparoscopic IPSDM, while no recurrence was observed in open IPSDM. Although no recurrence was observed in any patient in the open IPSDM, there was no statistically significant difference between the groups (p = 0.099).

Mortality due to medical complications was observed in a total of three (0.8%) patients. Mortality was observed in two patients (0.6.7%) in the open OPM and in one patient (0.25%) in the laparoscopic IPSDM, while no recurrence was observed in the open IPSDM. There was no statistically significant difference between the groups in mortality rates (p = 0.450) (Table 2).

**Table 2:** Postoperative complications * In the laparoscopic IPSDM method, two of the recurrences developed in the early period, and four of them developed in the late period. OPM, onlay prolene method; IPSDM, intraperitoneal sublay dual mesh method.

Complications	Patients	Methods of surgery			
	Total = 366	Open OPM (n = 300)	Laparoscopic IPSDM (n = 40)	Open IPSDM (n = 26)	p
Group 1: Wound complications					
1 - Seroma	47 (12.84%)	42 (14%)	4 (10%)	1 (3.85%)	0.360
2 - Bleeding	6 (1.66%)	5 (1.67%)	0	1 (3.85%)	0.455
3 - Superficial wound infection	26 (7.10%)	25 (8.33%)	0	1 (3.85%)	0.112
4 - Deep wound infection	2 (0.54%)	2 (0.67%)	0	0	1
5 - Skin erosion	11 (3.0%)	10 (3.33%)	0	1 (3.85%)	0.470
6 - Skin necrosis	2 (0.54%)	2 (0.67%)	0	0	1
7 - Mesh reaction	3 (0.81%)	3 (1%)	0	0	1
8 - Enterocutaneous fistula	1 (0.27%)	1 (0. 33%)	0	0	1
Total wound complications	98 (26.7%)	90 (30%)	4 (10%)	4 (15.3%)	0.009 (1-2, 1-3)
Group 2: Complications due to surgical procedure					
1 - Abdominal compartment syndrome	1 (0.27%)	1 (0.33%)	0	0	1
2 - Early mesh dehiscence	2 (0.54%)	0	2* (5%)	0	0,017
3 - Bowel injury	7 (1.91%)	5 (1.66%)	2 (5%)	0	0.245
4 - Early intra-abdominal bowel fistula	1 (0.27%)	0	1 (2.5%)	0	0.18
5 - Trocar site hernia	1 (0.27%)	0	1 (2.5%)	0	0.18
Total surgical complications	12 (3.2%)	6 (2%)	6 (15%)	0	0.002 (2-1, 2-3)
Group 3: Medical complications					
1 - Ileus	13 (3.55%)	10 (3.33%)	1 (2.5%)	2 (7.69%)	0.365
2 - Pulmonary atelectasis	7 (1.91%)	5 (1.67%)	1 (2.5%)	1 (3.85%)	0.369
3 - Pulmonary embolism	1 (0.27%)	1 (0.33%)	0	0	1
4 - Deep vein thrombosis	2 (0.54%)	2 (0.67%)	0	0	1
5 - Myocardial infarction	1 (0.27%)	1 (0.33%)	0	0	1
Total medical complications	24 (6.5%)	19 (6.3%)	2 (5%)	3 (11.5%)	0.540
Group 4: Number of recurrences	34 (9.2%)	28 (9.3%)	6 (4+2*) (15%)	0	0.099
Group 5: Mortality	3 (0.81%)	2 (0.67%)	1 (2.5%)	0	0.450
Number of total complications	169 (46%)	145 (48.3%)	17 (42.5%)	7 (27%)	0.092

## Discussion

Wound complications are the most common morbidity after LIH repair. Infections, seromas, hematomas, and skin erosions are common after LIH repairs. There is a large difference between the surgical methods used and wound complications. Wound complications vary from 13% to 48%, depending on the surgical technique used [1,8]. In our study, wound complications occurred in 98 (26.7%) patients. Depending on the group, wound complications occurred in 90 patients (30%) in the open OPM method, four patients (10%) in the laparoscopic IPSDM method, and four patients (15.3%) in the open IPSDM method. When the treatment methods were compared in terms of the incidence of seroma, hemorrhage, superficial wound infection, deep wound infection, skin erosion, skin necrosis, mesh reaction, and enterocutaneous fistula reported as wound complication types, no statistical difference was found (p > 0.05) (Table 2). However, considering the total number of patients who experienced wound complications, it was found that the complication rate was higher for the open OPM method compared to the other methods (p = 0.009). It was suggested that the wide detachment of skin and fascia for mesh placement might be why wound complications were more common with the open OPM method.

Complications related to the surgical procedure occurred in a total of 12 patients. There were six patients (2%) in the open OPM method, six patients (15%) in the laparoscopic IPSDM method, and no surgical complications were observed in the open IPSDM method. In the laparoscopic IPSDM method, early mesh detachment after surgery was statistically significant in two patients (p = 0.017). When the complication rate due to the entire surgical procedure was compared, it was found that the surgical complication rate was statistically higher for the laparoscopic IPSDM method than for the other two methods (p = 0.002). Two reasons were effective for the high rate of surgical complications with the laparoscopic IPSDM method. First, detachment of the mesh in the initial stage due to the more difficult positioning of the mesh in the laparoscopic method; second, unrecognized bowel injury was found to be the cause (Table 2).

In group 3, medical complications occurred in 24 (6.5%) patients. There was no significant difference between the groups in the rate of medical complications (p = 0.054). To avoid medical complications in treating LIH, preventive prophylactic treatments against pulmonary atelectasis and thromboembolic events should be initiated, especially in patients with high comorbidities and high BMI, and patients should be monitored closely.

The main criterion for the success of an LIH repair is the recurrence rate [15]. According to the groups, recurrence was observed in 28 patients (9.3%) in the open OPM method, and in six (15%) patients in the laparoscopic IPSDM method, whereas no recurrence was observed in the open IPSDM method. When the surgical treatment methods were compared, no statistically significant difference was found between the groups (p = 0.099). However, it was noticeable in the open IPSDM method that the hernia defect was larger, but no recurrence was observed (Table 2). A meta-analysis study conducted in the literature reported that sublay position mesh placement was less recurrent in the long term [1,17]. There are two aspects to the treatment of LIH. Proper placement of the mesh at the defect margins to completely cover the defect and strong fixation of the mesh to the abdominal wall. Sublay mesh is used in both the open IPSDM and the laparoscopic IPSDM methods. However, the lack of recurrence in the onlay IPSDM method was thought to be a fixation with more prolene sutures and better positioning of the mesh at the defect margins. Information on how the surgical technique is applied in the open IPSDM method was explained in our previous article [18]. Mortality in small incisional hernias is usually due to medical problems such as cardiovascular and pulmonary problems in patients with high comorbidity. Mortality in patients with complex LIHs is associated with wound complications, wound infections, mesh infections, sepsis, and multiorgan failure. In a study by Basta et al., 80% of patients who experienced mortality had medical complications, whereas mortality occurred in 20% because of surgical complications [19]. In our patient group, mortality occurred in three patients. In two patients operated on with the open OPM method, mortality was due to cardiovascular and pulmonary problems. One patient with the laparoscopic IPSDM method died from bowel perforation and sepsis. The mortality rate was reported to be 0.8%, which is consistent with the literature.

Some studies reported a shorter hospital stay for the laparoscopic method [20]. In our study, no significant difference in hospital stay was found in all three methods (p* *= 0.43). In our study, it was thought that the average hospital stay was increased due to the complications of early mesh detachment (n = 2) and bowel injury (n = 3) in the laparoscopic IPSDM method. In a study by Cox et al., complications after incisional hernia surgery were reported to increase the length of hospital stay [13].

In a study conducted by Al Chalabi et al., it was found that the operative time was longer in the group of patients who underwent laparoscopic incisional hernia, although this was not statistically significant [21]. In our study, it was found statistically significant that the duration of operation was shorter in the open IPSDM method compared to both the open OPM method and the laparoscopic IPSDM method in terms of mean duration of operation (p < 0.001). It was suggested that the operative time was shorter with the open IPSDM method than with the open OPM method because the fascia was not detached extensively. Because of technical difficulties, such as positioning the mesh in the abdomen in the laparoscopic IPSDM method, it was assumed that the average duration of operation was longer than the open IPSDM method.

The main limitation of our study is that it is a retrospective study. Selection bias may lead to bias in the choice of treatment method. Despite these limitations, we believe that our study has certain strengths. Considering the number of patients analyzed, our study is a large series. It is a unique study in terms of investigating early complications and recurrence risk according to the type of surgical method to be applied. It will contribute to the studies on the morbidity rates and recurrence rates in the selection of the surgical treatment type for LIHs. Prospective randomized clinical and technical studies with similar groups of patients are needed to obtain more accurate results.

## Conclusions

Although the hernia defect was greater with the open IPSDM method compared with other methods, it was more beneficial than other methods in terms of the complication rate associated with the surgical procedure, the overall complication rate, the operative time, and the recurrence rate. The overall wound complication rate of laparoscopic IPSDM is a more beneficial method in terms of medical complications. Although open OPM is the most commonly used method, it has the highest hospital length of stay and overall complication rate.

However, there seems to be a need for high-quality, evidence-based studies from large-scale randomized trials to obtain more meaningful results.

## References

[1] Deerenberg EB, Timmermans L, Hogerzeil DP, Slieker JC, Eilers PH, Jeekel J, Lange JF (2015). A systematic review of the surgical treatment of large incisional hernia. Hernia.

[2] Eriksson A, Rosenberg J, Bisgaard T (2014). Surgical treatment for giant incisional hernia: a qualitative systematic review. Hernia.

[3] Cornette B, De Bacquer D, Berrevoet F (2018). Component separation technique for giant incisional hernia: a systematic review. Am J Surg.

[4] Kumar SJG, Kumar UK, Manangi M, Madhu KP, Arun BJ, Nagaraj N (2016). Incisional hernia: incidence, clinical profile, risk factors and prevention. Int Surg J.

[5] Walming S, Angenete E, Block M, Bock D, Gessler B, Haglind E (2017). Retrospective review of risk factors for surgical wound dehiscence and incisional hernia. BMC Surg.

[6] Muysoms FE, Miserez M, Berrevoet F (2009). Classification of primary and incisional abdominal wall hernias. Hernia.

[7] Chevrel JP, Rath AM (2000). Classification of incisional hernias of the abdominal wall. Hernia.

[8] Memon AA, Khan A, Zafar H, Murtaza G, Zaidi M (2013). Repair of large and giant incisional hernia with onlay mesh: perspective of a tertiary care hospital of a developing country. Int J Surg.

[9] Helgstrand F, Rosenberg J, Kehlet H, Jorgensen LN, Bisgaard T (2013). Nationwide prospective study of outcomes after elective incisional hernia repair. J Am Coll Surg.

[10] Kroese LF, Kleinrensink GJ, Lange JF, Gillion JF (2018). External validation of the European Hernia Society classification for postoperative complications after incisional hernia repair: a cohort study of 2,191 patients. J Am Coll Surg.

[11] Lindmark M, Strigård K, Löwenmark T, Dahlstrand U, Gunnarsson U (2018). Risk factors for surgical complications in ventral hernia repair. World J Surg.

[12] Breuing K, Butler CE, Ferzoco S (2010). Incisional ventral hernias: review of the literature and recommendations regarding the grading and technique of repair. Surgery.

[13] Cox TC, Blair LJ, Huntington CR (2016). The cost of preventable comorbidities on wound complications in open ventral hernia repair. J Surg Res.

[14] Holmdahl V, Stark B, Clay L, Gunnarsson U, Strigård K (2022). Long-term follow-up of full-thickness skin grafting in giant incisional hernia repair: a randomised controlled trial. Hernia.

[15] Bittner R, Bain K, Bansal VK (2019). Update of guidelines for laparoscopic treatment of ventral and incisional abdominal wall hernias (International Endohernia Society (IEHS)): part B. Surg Endosc.

[16] de Vries Reilingh TS, van Goor H, Charbon JA, Rosman C, Hesselink EJ, van der Wilt GJ, Bleichrodt RP (2007). Repair of giant midline abdominal wall hernias: "components separation technique" versus prosthetic repair: interim analysis of a randomized controlled trial. World J Surg.

[17] Timmermans L, de Goede B, van Dijk SM, Kleinrensink GJ, Jeekel J, Lange JF (2014). Meta-analysis of sublay versus onlay mesh repair in incisional hernia surgery. Am J Surg.

[18] Korkut E, Aksungur N, Altundaş N, Kara S, Peksöz R, Öztürk G (2022). Giant incisional hernia repair using open intraperitoneal dual mesh. Cureus.

[19] Basta MN, Fischer JP, Wink JD, Kovach SJ (2016). Mortality after inpatient open ventral hernia repair: developing a risk stratification tool based on 55,760 operations. Am J Surg.

[20] Ross SW, Wormer BA, Kim M (2015). Defining surgical outcomes and quality of life in massive ventral hernia repair: an international multicenter prospective study. Am J Surg.

[21] Al Chalabi H, Larkin J, Mehigan B, McCormick P (2015). A systematic review of laparoscopic versus open abdominal incisional hernia repair, with meta-analysis of randomized controlled trials. Int J Surg.

